# Genetic diversity of human head lice and molecular detection of associated bacterial pathogens in Democratic Republic of Congo

**DOI:** 10.1186/s13071-019-3540-6

**Published:** 2019-06-07

**Authors:** Celia Scherelle Boumbanda Koyo, Nadia Amanzougaghene, Bernard Davoust, Leon Tshilolo, Jean Bernard Lekana-Douki, Didier Raoult, Oleg Mediannikov, Florence Fenollar

**Affiliations:** 1 Aix Marseille Univ, IRD, AP-HM, SSA, VITROME, Marseille, France; 2 Aix Marseille Univ, IRD, AP-HM, MEPHI, Marseille, France; 3 IHU-Méditerranée Infection, Marseille, France; 4 Monkole Mother and Child Hospital, Kinshasa, Democratic Republic of the Congo; 5 Unité d’Evolution Epidémiologie et Résistances Parasitaires (UNEEREP), Centre International de Recherches Médicales de Franceville (CIRMF), Franceville, Gabon; 6 Ecole Doctorale Régionale en Infectiologie Tropicale d’Afrique Centrale, Franceville, Gabon; 7 Département de Parasitologie-Mycologie Médecine Tropicale, Faculté de Médecine, Université des Sciences de la Santé (USS), Libreville, Gabon

**Keywords:** Head lice, Clade E, *Acinetobacter baumannii*, *Acinetobacter* spp., Democratic Republic of Congo

## Abstract

**Background:**

Head louse, *Pediculus humanus capitis*, is an obligatory blood-sucking ectoparasite, distributed worldwide. Phylogenetically, it occurs in five divergent mitochondrial clades (A–E); each exhibiting a particular geographical distribution. Recent studies suggest that, as in the case of body louse, head louse could be a disease vector. We aimed to study the genetic diversity of head lice collected in the Democratic Republic of the Congo (DR Congo) and to screen for louse-borne pathogens in these lice.

**Methods:**

A total of 181 head lice were collected from 27 individuals at the Monkole Hospital Center located in Kinshasa. All head lice were genotyped and screened for the presence of louse-borne bacteria using molecular methods. We searched for *Bartonella quintana*, *Borrelia recurrentis*, *Rickettsia prowazekii*, *Anaplasma* spp., *Yersinia pestis*, *Coxiella burnetii* and *Acinetobacter* spp.

**Results:**

Among these head lice, 67.4% (122/181) belonged to clade A and 24.3% (44/181) belonged to clade D. Additionally, for the first time in this area, we found clade E in 8.3% (15/181) of tested lice, from two infested individuals. Dual infestation with clades A and D was observed for 44.4% individuals. Thirty-three of the 181 head lice were infected only by different bacterial species of the genus *Acinetobacter*. Overall, 16 out of 27 individuals were infested (59.3%). Six *Acinetobacter* species were detected including *Acinetobacter baumannii* (8.3%), *Acinetobacter johnsonii* (1.7%), *Acinetobacter soli* (1.7%), *Acinetobacter pittii* (1.7%), *Acinetobacter guillouiae* (1.1%), as well as a new potential species named “*Candidatus* Acinetobacter pediculi”.

**Conclusions:**

To our knowledge, this study reports for the first time, the presence of clade E head lice in DR Congo. This study is also the first to report the presence of *Acinetobacter* species DNAs in human head lice in DR Congo.

**Electronic supplementary material:**

The online version of this article (10.1186/s13071-019-3540-6) contains supplementary material, which is available to authorized users.

## Background

Two lice species infested humans: *Pediculus humanus* and *Pthirus pubis* [1]. The first is of great public health concern and includes two ecotypes: *Pediculus humanus capitis* head lice, which live in the scalp area, and *Pediculus humanus humanus* body lice, which live in clothing [1, 2].

Studies based on mitochondrial genes appear to separate head and body lice into five divergent clades (A, B, C, D and E) exhibiting some geographical differences [3–5]. Head lice encompass all diversity while body lice belong only to clades A and D [3, 6]. It is well known that only clade A has a worldwide distribution [1]. However, with the globalization, clades B to E tend to disperse throughout the world. Originally, Clade B was found in Europe and in the New World [3], Clade C in Africa and Asia [7], Clade D in the Democratic Republic of Congo (DR Congo) [5], and Clade E in West Africa (Mali) [6]. Based on archeological remains, the *Pediculus* louse is thought to be an ancient parasite that had long association with their human hosts [8]. Because of this long association lice have become a model for studying the cophylogenetic relationships between hosts and parasites [9].

Although it is currently assumed that body lice are more potent vectors of pathogens, the potential role of head lice as a vector is not fully understood. Studies have shown that the immune responses of head lice to different pathogens are stronger than those of body lice, which obviously can carry a broad spectrum of pathogens [10, 11]. In recent decades, the DNA of several pathogenic bacteria has been increasingly detected in head lice collected around the world. This is the case of *Bartonella quintana*, *Borrelia recurrentis*, *Yersinia pestis*, *Borrelia theileri*, *Coxiella burnetii*, *Rickettsia aeschlimannii*, as well as of potential new species of the genera *Anaplasma* and *Ehrlichia* detected in head lice belonging to different mitochondrial clades [5, 6, 12–17]. Several species of *Acinetobacter*, including potential new species, have also been detected in human head lice [12, 18–20]. In addition, experimental infections with *R. prowazekii* have shown that head lice can be easily infected and spread these pathogens in their feces, demonstrating that these lice could have the potential to be vectors of pathogens under optimal epidemiological conditions [21]. In laboratory-reared lice, it has been shown that head lice can support a persistent load of *B. quintana* infection for several days following acquisition in a blood meal and disseminate viable organisms in their feces [10, 11]. This fact poses a very substantial health risk for infested persons because head lice infestations are widespread around the world and epidemics still occur regularly. Children are at increased risk, regardless of hygiene conditions and social status [22].

In this study, we aimed to investigate the genetic diversity of head lice collected in the DR Congo and to look for pathogenic bacteria in these lice.

## Methods

### Lice collection

Lice collection was carried out at the medical center of Monkole located at Kinshasa, the largest city and capital of the DR Congo. In total, 27 patients were enrolled and thoroughly examined for detection of both body and head lice. They came from 11 geographically very close communities. A total of 181 head lice were collected from these patients. No body lice were found during the examination. The lice collected were preserved in 70% alcohol and were then sent to the Laboratory of IHU-Méditerranée Infection, Marseille, France, stored at room temperature.

### DNA extraction

To avoid bacterial contamination of lice external surface, each louse specimen was decontaminated, as described previously [23], and rinsed twice in distilled sterile water. Then, each louse was dried and cut in half lengthwise. Half was frozen at −20 °C for later use. The remaining half was crushed in sterile Eppendorf tube; total DNA was extracted using a DNA extraction kit, QIAamp Tissue Kit (Qiagen, Courtaboeuf, France) in the EZ1 apparatus following the manufacturer’s protocol. The DNA was eluted in 100 μl of TE (10/1) buffer and stored at 4 °C until used for PCR amplifications. DNA quantity and quality were assessed using a NanoDrop ND-1000 (Thermo Fisher Scientific, Waltham, MA, USA).

### Genotypic status of lice

#### Identification of louse mitochondrial clade by qPCR assays

To identify the mitochondrial clades of the lice included in this study, all DNA samples were analyzed using clade-specific quantitative real-time PCR (qPCR) assays that targeted a portion of *cytochrome b* (*cytb*) gene specific to each of the five clades, as previously described [6]. We used lice with known clades as positive controls and master mixtures as negative control for each assay. All PCR amplifications were carried out using a CFX96 Real-Time system (Bio-Rad Laboratories, Foster City, CA, USA), as previously described [6].

#### Cytochrome b amplification and haplotype determination

For the phylogenetic study, DNA samples of 54 head lice randomly selected from the total number of lice, were subjected to standard PCR targeting a 347-bp fragment of the *cytb* gene, using the primers and conditions previously described [24]. The PCR consisted of a 50 µl volume, including 25 µl Amplitaq gold master mixes, 1 µl of each primer, 5 μl DNA template, and water. The thermal cycling profile was one incubation step at 95 °C for 15 min, 40 cycles of 1 min at 95 °C, 30 s at 56 °C and 1 min at 72 °C, followed by a final extension step for 5 min at 72 °C.

PCR amplification was performed in a Peltier PTC-200 model thermal cycler (MJ Research Inc, Watertown, MA, USA). The success of amplification was confirmed by electrophoresis on agarose gel. The purification of PCR products was performed using NucleoFast 96 PCR plates (Macherey-Nagel EURL, Hoerdt, France) according to the manufacturer’s instructions. The amplicons were sequenced using the Big Dye Terminator Cycle Sequencing Kit (Perkin Elmer Applied Biosystems, Foster City, CA) with an ABI automated sequencer (Applied Biosystems). The electrophoregrams obtained were assembled and edited using the ChromasPro software (ChromasPro 1.7, Technelysium Pty Ltd., Tewantin, Australia).

### Molecular screening for the presence of bacterial DNA

The qPCRs were performed to screen all lice samples, using previously reported primers and probes for *Borrelia* spp., *B. quintana*, *Acinetobacter* spp., *Rickettsia* spp., *R. prowazekii*, *Y. pestis*, *Anaplasma* spp. and *C. burnetii*. Sequences of primers and probes are shown in Table 1 [6, 12, 13, 18, 24–29].

**Table 1:** Oligonucleotide sequences of primers and probes used for real-time PCRs and conventional PCRs in this study

Target	Name	Sequence (5′–3′) and probes	Source
*P. humanus* *Cytochrome b*	Duplex A–D	F: GATGTAAATAGAGGGTGGTT	[6, 12]
		R: GAAATTCCTGAAAATCAAAC	
		FAM-CATTCTTGTCTACGTTCATATTTGG-TAMRA	
		VIC-TATTCTTGTCTACGTTCATGTTTGA-TAMRA	
	Duplex B–C/E	F: TTAGAGCGMTTRTTTACCC	
		R: AYAAACACACAAAAMCTCCT	
		FAM-GAGCTGGATAGTGATAAGGTTTAT-MGB	
		VIC-CTTGCCGTTTATTTTGTTGGGGTTT-TAMRA	
	Monoplex E	F: GGTTGGAATTGGATAGTGAT	
		R: GGGTCCATAAAGAAATCCG	
		FAM- TAGGAGGCTTTGTGTGTCTATCCT-TAMRA	
	*Cytb*	F: GAGCGACTGTAATTACTAATC	[24]
		R: CAACAAAATTATCCGGGTCC	
*Acinetobacter* spp. RNA polymerase β subunit gene	*rpoB*	F: TACTCATATACCGAAAAGAAACGG	[18]
		R: GGYTTACCAAGRCTATACTCAAC	
		FAM-CGCGAAGATATCGGTCTSCAAGC-TAMRA	
	*rpoB* (zone1)	F: TAYCGYAAAGAYTTGAAAGAAG	[25]
		R: CMACACCYTTGTTMCCRTGA	
*R. prowazekii* *rOmpB* gene	*ompB*	F: AATGCTCTTGCAGCTGGTTCT	
		R: TCGAGTGCTAATATTTTTGAAGCA	
		FAM-CGGTGGTGTTAATGCTGCGTTACAACA-TAMRA	
*Y. pestis* Plasminogen activator gene	*PLA*	F: ATGGAGCTTATACCGGAAAC	[26]
		R: GCGATACTGGCCTGCAAG	
		FAM-TCCCGAAAGGAGTGCGGGTAATAGG-TAMRA	
*Borrelia* spp. *16S* ribosomal RNA	*Bor16S*	F: AGCCTTTAAAGCTTCGCTTGTAG	[27]
		R: GCCTCCCGTAGGAGTCTGG	
		FAM-CCGGCCTGAGAGGGTGAACGG-TAMRA	
*B. quintana* Hypothetical intracellular effector	*yopP*	F: TAAACCTCGGGGGAAGCAGA	[13]
		R: TTTCGTCCTCAACCCCATCA	
		FAM-CGTTGCCGACAAGACGTCCTTG-TAMRA	
3-oxoacyl-synthase gene	*fabF3*	F: GCGGCCTTGCTCTTGATGA	
		R: GCTACTCTGCGTGCCTTGGA	
		FAM-TGCAGCAGGTGGAGAGAACGTG-TAMRA	
*Anaplasma* spp. *23S* ribosomal RNA	TtAna	F: TGACAGCGTACCTTTTGCAT	[28]
		R: TGGAGGACCGAACCTGTTAC	
		FAM-GGATTAGACCCGAAACCAAG-TAMRA	
*C. burnetii* IS1111 spacer	IS1111	F: CAAGAAACGTATCGCTGTGGC	[29]
		R: CACAGAGCCACCGTATGAATC	
		FAM-CCGAGTTCGAAACAATGAGGGCTG-TAMRA	

The qPCRs were performed using a CFX96 Real-Time system (Bio-Rad) and the Roche LightCycler 480 Probes Master Mix PCR kit (Roche Applied Science, Mannheim Germany). We included DNA extracts of the targeted bacteria as positive controls and master mixtures as negative control for each assay. We considered samples to be positive when the cycle’s threshold (Ct) was lower than 35 Ct [30].

In order to identify the species of *Acinetobacter*, all positive samples from qPCR were subjected to standard PCR, targeting a portion of the *rpoB* gene (zone 1) using primers and conditions previously described [25]. Successful amplification was confirmed *via* gel electrophoresis and amplicons were prepared and sequenced using similar methods as described for the *cytb* gene for lice above.

### Data analysis

Unique haplotypes were defined by using DnaSPv5.10 to obtain head lice *cytb* sequences, then, compared and combined (Additional file 1: Table S1), with the *cytb* haplotypes previously reported [12]. In order to investigate the possible relationships between the haplotypes, the median-joining (MJ) network using the method of Bandelt was constructed with the program NETWORK4.6 (http://www.fluxus-engineering.com/sharenet.htm) using equal weights for all mutations [31]. Phylogenetic analyses and tree reconstruction were performed using MEGA software v.6.06 [32]. Phylogenetic analysis was performed using maximum likelihood (ML) approach. To generate the best ML tree, Modeltest v.3.7 [33] was used to examine model of nucleotide substitution and choose a best-fit model of sequence evolution. The model that provides the best approximation of the data using the fewest parameters was chosen for the analysis according to the Akaike information criterion [34, 35]. Tree reconstruction was conducted using MEGA software v.6.06 under HKY+I+G model with 500 bootstrap replicates. All obtained sequences of *Acinetobacter* spp. were analyzed using BLAST (www.ncbi.nlm.nih.gov/blast/Blast.cgi) and compared with sequences in the GenBank database. A maximum-likelihood method was used to infer the phylogenetic analyses and the best-fit model was chosen as described for *cytb* sequences above. The tree reconstruction was performed using the TrN+ G model for nucleotide sequences under 500 bootstrap replicates in MEGA software v.6.06 [32].

## Results

### Lice clade and phylogenetic analyses

Overall, 181 head lice were collected from 27 obviously healthy women. The average number of lice per individual was 6.7 ± 6.6. All collected lice were tested by qPCRs to determine their clade. Our results showed that 67.4% (122/181) of lice belonged to clade A, 24.3% (44/181) to clade D, and only 8.3% (15/181) belonged to clade E. Among the 27 persons, 15 (55.6%) were infested by only one clade. Among them, 8 (29.6%) individuals were only infested with clade A, 5 (18.5%) only with clade D, and 2 (7.4%) only with clade E. Finally, dual infestation was observed in 12 individuals (44.4%), and only with clades A and D (Table 2).

**Table 2:** Number of infested people by one or more clades of lice in this study

Clade of lice	People infested (*n* = 27)	
	*n*	%
Single infestation		
Clade A	8	29.63
Clade D	5	18.52
Clade E	2	7.41
Total	15	55.56
Multiple infestation		
Clade A/D	12	44.44
Clade A/E	0	0
Clade D/E	0	0
Clade A/D/E	0	0
Total	12	44.44

The analysis of a 347-bp fragment of 54 *cytb* sequences yielded 43 variable positions defining 10 different haplotypes. One haplotype belonged to the worldwide haplotype A5 within clade A. Four haplotypes also belonging to clade A, were new and were named A66-A69 (Table 3).

**Table 3:** Haplotype frequency of head and body lice identified in 54 head lice

Clade of lice	Haplotype	*n*	GenBank ID
Clade A	A5	16	KM579542
	A66	7	**MH230928**
	A67	2	**MH230927**
	A68	2	**MH230926**
	A69	1	**MH230925**
Clade D	D60	3	KX249766
	D74	4	**MH230924**
	D75	1	**MH230923**
	D76	8	**MH230922**
Clade E	E62	10	**MH230921**
Total		54	

*Note*: The new haplotypes identified in this study are in bold

**Fig. 1:**
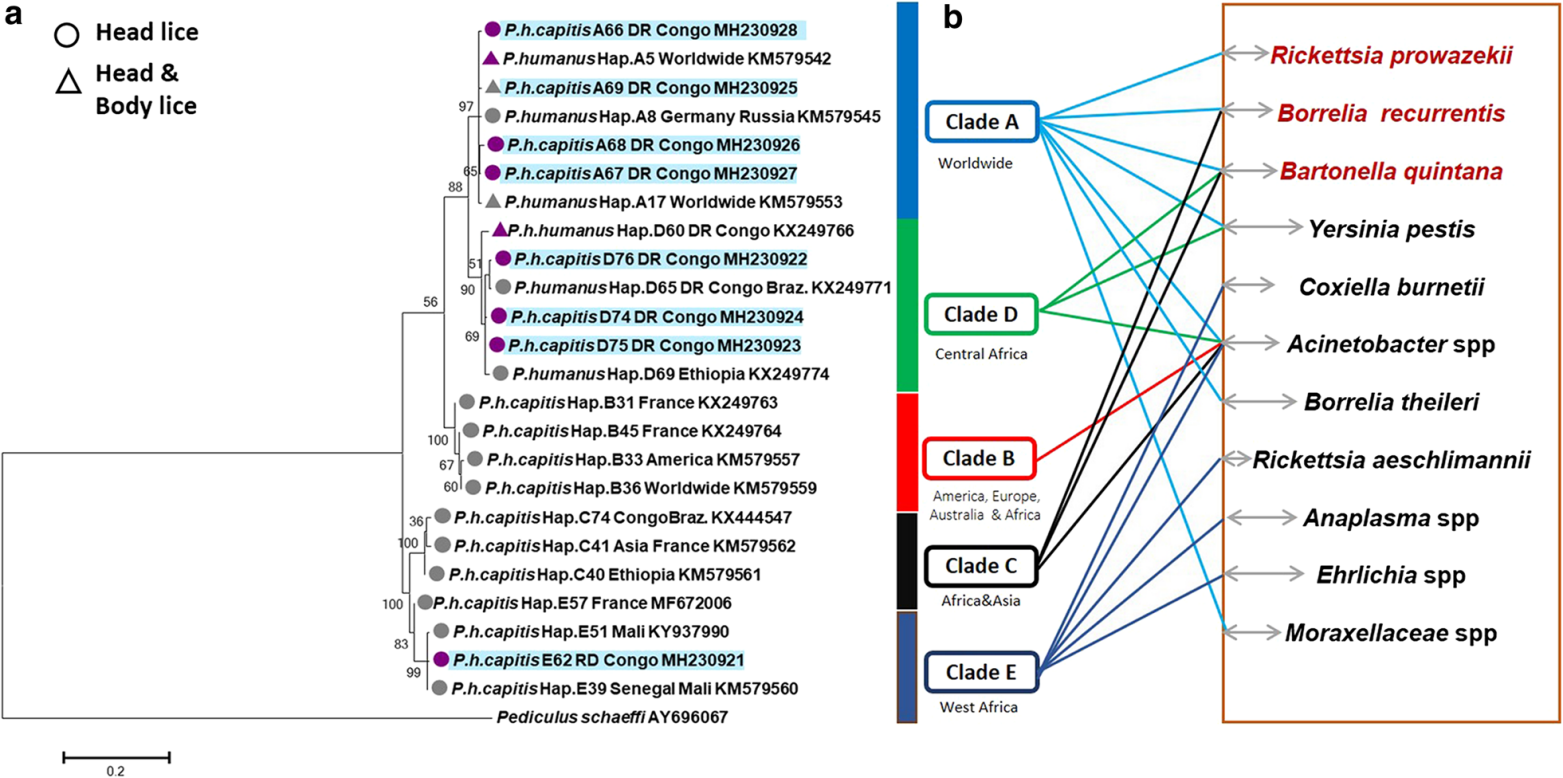
Maximum-likelihood (ML) phylogram of the mitochondrial *Cytb* haplotypes. **a** Phylogenetic inference was conducted in MEGA 6 using the maximum likelihood method under HKY + I + G model with 500 bootstrap replicates. The novel haplotypes identified in this study are indicated in blue. **b** Bacterial DNAs detected in head lice reported in this study and the literature. The pathogenic bacteria in red are those naturally transmitted by body lice to humans

**Fig. 2:**
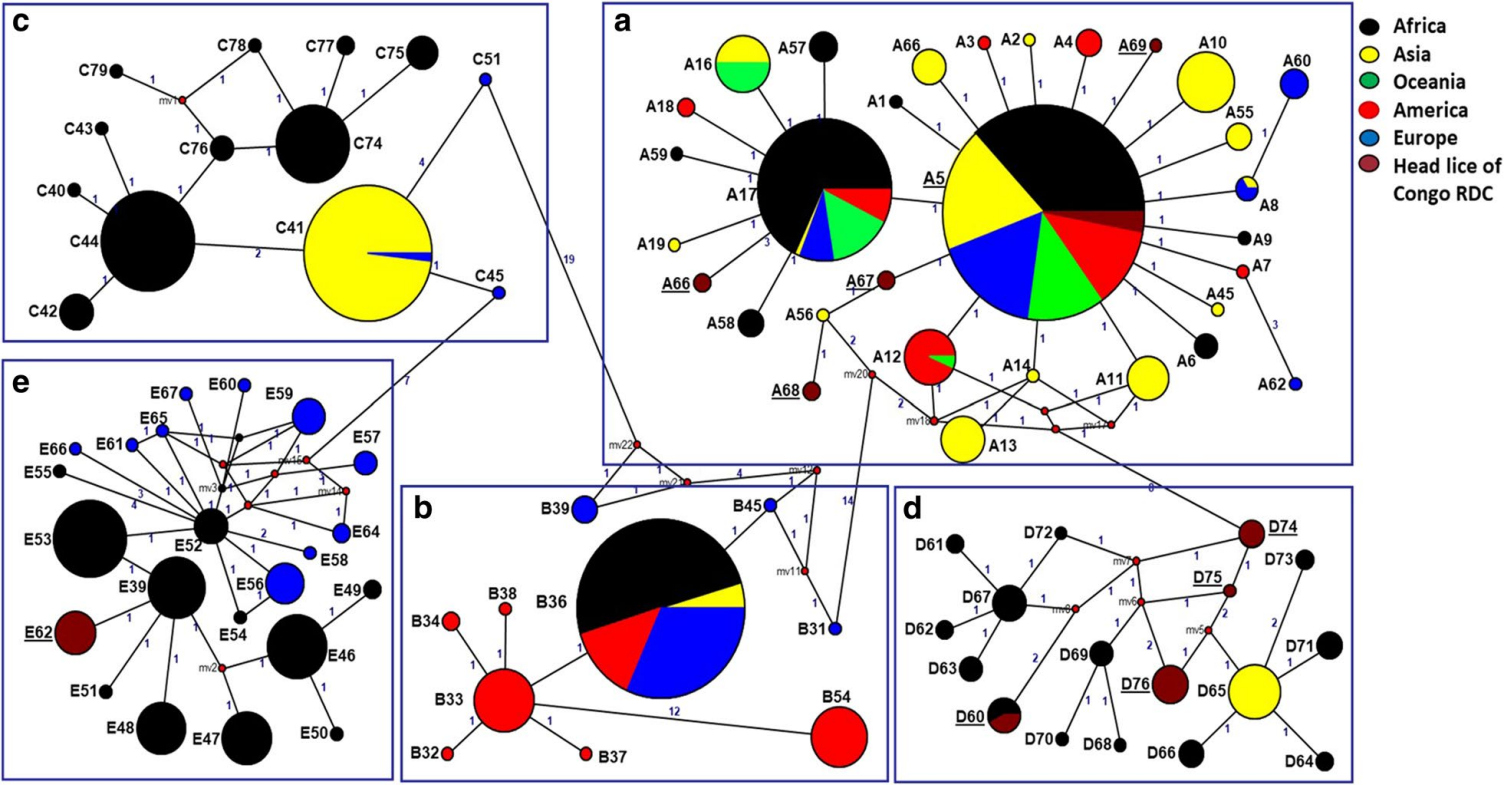
*Cytb* haplotype networks of human body and head lice. The five connected subnets corresponding to the known clades: A, D, B, C and E. Each circle indicates a unique haplotype, and variations in circle size are proportional to haplotype frequencies. Pie colors and sizes in circles represent the continents and the number of their sequence for a haplotype. The length of the links between nodes is proportional to the number of mutations. The types of haplotypes identified in this study are underlined

Within clade D, four haplotypes were identified; one of them belonged to haplotype D60. The other three haplotypes were novel and are referred to as D74-D76. The remaining haplotype belonging to clade E was novel and is referred to as E62 (Table 3). Analyses of the phylogenetic tree and the median assemblage yield similar results; all *cytb* sequences were distributed among five major supported clades, represented by five connected subnets and separate groups as shown by the MJ network corresponding to the known clades: A, D, B, C and E. The 10 haplotypes of our study fell into the three clades A, D and E (Figs. 1, 2). All sequences of *cytb* haplotypes of *P. h. capitis* obtained, in this study, were deposited in the GenBank database, under the accession numbers MH230921-MH230928.

### Molecular detection of bacterial DNA

All the head lice tested on qPCRs were negative for *B. quintana*, *Y. pestis*, *C. burnetii*, *Borrelia*, *Anaplasma*, *Rickettsia* spp. and *R. prowazekii*. The DNA of *Acinetobacter* spp. was detected in 33 of the 181 head lice (18.2%), infesting 16 of 27 individuals (59.3%). Sequencing of 350-bps fragment *rpoB* gene coupled with blast analysis revealed that 26 of 33 sequences (78.8%) match five species of *Acinetobacter* sharing 99–100% identity with their corresponding reference *Acinetobacter* spp., namely *A. baumannii*, *A. pittii*, *A. soli*, *A. guillouiae* and *A. johnsonii. Acinetobacter baumannii* was the most frequently identified species with a prevalence of 8.3%, followed by *A. pittii*, *A. soli* and *A. johnsonii* (1.7%), then *A. guillouiae* (1.1%). For two of the 33 sequences, BLAST analysis showed a homology score lower than 95%, meaning that these sequences are likely to correspond to new species, provisionally referred to here as “*Candidatus* Acinetobacter pediculii”. The most closely-related species is *A. guillouiae* (GenBank: FJ754439) with 94.9% similarity (337 of 355 base positions in common).

**Fig. 3:**
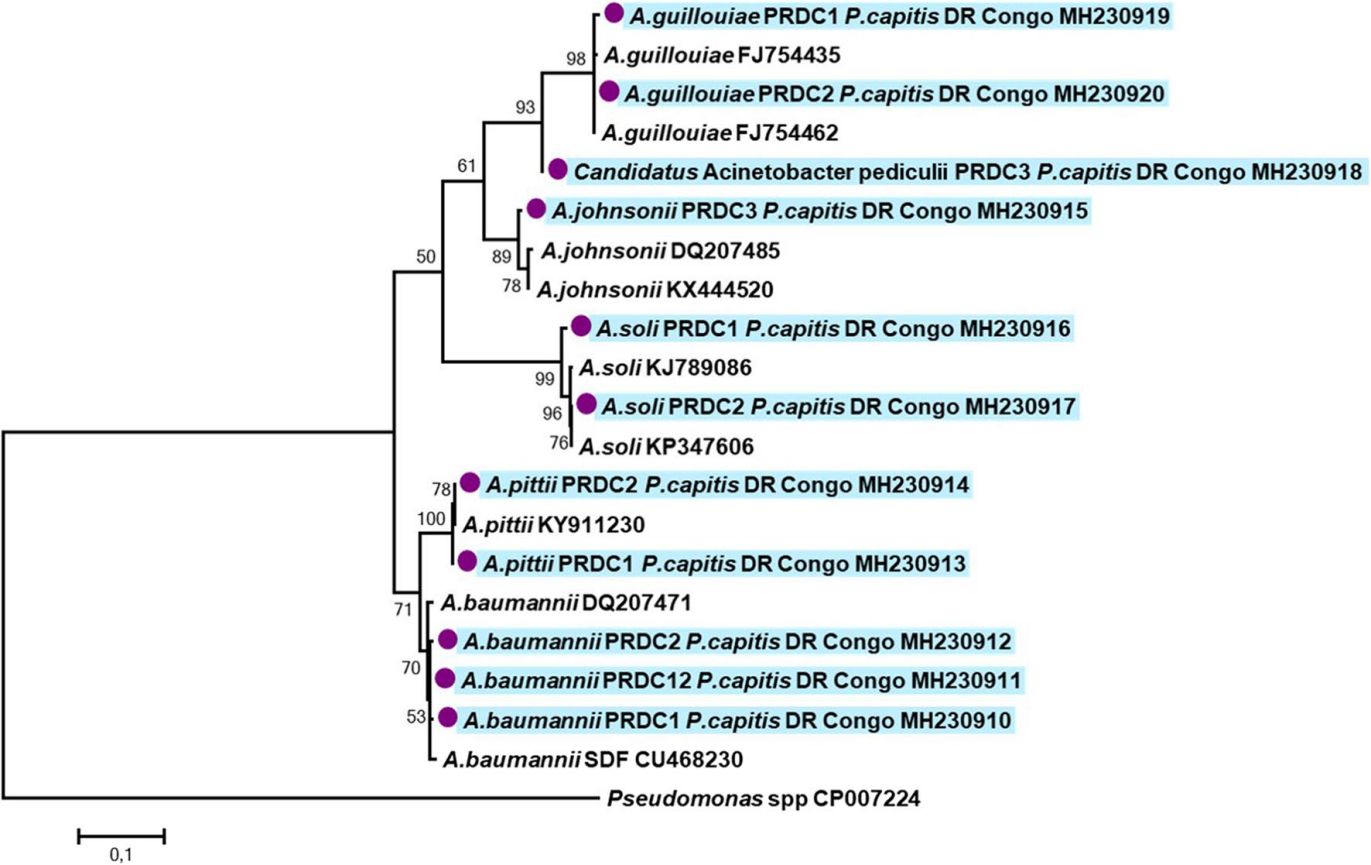
Phylogenetic tree highlighting the position of *Acinetobacter* spp. identified in head lice from DR Congo. The *rpoB* sequences were aligned using CLUSTALW, and phylogenetic inferences were conducted in MEGA 6 using the maximum likelihood method based on the TrN + G model for nucleotide sequences. Statistical support for internal branches of the trees was evaluated by bootstrapping with 500 iterations. There was a total of 345 positions in the final dataset

The remaining five of 33 sequences (15.1%) presented also some similarities with *Acinetobacter*. However, the sequences were of poor quality, which is assumed to be due to co-infection with several *Acinetobacter* species. The distribution of *Acinetobacter* species according to lice clades are presented in Table 4. The phylogenetic positions of all *Acinetobacter* species identified in this study are presented in Fig. 3. The partial *rpoB* sequences obtained in this study were deposited in the GenBank database under the accession numbers: MH230910-MH230920.

**Table 4:** Summary of bacterial species detected in head lice collected from infested individuals in DR Congo per lice clade

	Number of positive for *Acinetobacter* spp. (%)			
	Clade A (*n* = 122)	Clade D (*n* = 44)	Clade E (*n* = 15)	Total (*n* = 181)
*A. baumannii*	8 (6.5)	5 (11.4)	2 (13.3)	15 (8.3)
*A. soli*	3 (2.4)	0	0	3 (1.7)
*A. pittii*	2 (1.6)	1 (2.3)	0	3 (1.7)
*A. johnsonii*	2 (1.6)	1 (2.3)	0	3 (1.7)
*A. guillouiae*	2 (1.6)	0	0	2 (1.1)
“*Candidatus* A. pediculi”	2 (1.6)	0	0	2 (1.1)
*Acinetobacter* spp.	1 (0.8)	1 (2.3)	3 (20)	5 (2.8)
Total	20 (16.4)	8 (18.2)	5 (33.3)	33 (18.2)

## Discussion

In this study, we investigated the genetic diversity of head lice collected in DR Congo. The presence of lice from clades A, D and E was observed. The most prevalent clade was A, confirming its worldwide distribution, followed by clade D and clade E. Clades A and D were already reported in this area [5]. But this is the first report of clade E in Central Africa, which is more abundant in West Africa [6]. All positive lice for clade E arise from only two individuals. Several hypotheses could be suggested, such as the recent arrival of these people from West African countries, close contact with West African populations, or a previous implantation of these low-prevalence lice. However, only one haplotype for all clade E lice was observed. This clade, named here E62, had never been described before in West Africa. Overall, these data show that the current repartition of human lice clades is not definitive. Increasing the samples sizes and extending the geographical coverage are needed to better determine the intra- and interclade diversity [3].

In addition to the inter-haplogroup diversity, *P. humanus* also showed intra-haplogroup diversity, which is illustrated by numerous distinct A, B, D, E and C haplotypes [3, 7, 12, 36], results supported by our findings. Indeed, among the 54 head lice *cytb* sequences analyzed, ten different haplotypes were identified; in which eight haplotypes were novel. There are several reports stating that co-infestation by different mitochondrial DNA (mtDNA) clades of human lice in the same individual can occur, and it was found to be associated with clades A and B [37, 38], clades A and C [38, 39] and also for clades A and D [5], suggesting that these different clades can live in sympatry and interbreed [37, 39]. Indeed, several studies have shown evidence of recombination events that occur between different lice clades living in sympatric by using intergenic spacers [37, 39]. Moreover, louse females have lost their spermatheca and must mate before laying eggs; frequent mating is essential, and this process encourages outbreeding [40].

In our study, only half of the individuals were mono-infested by one clade of lice. Dual infestation was observed only with clades A and D in 12 individuals (44%). These data are consistent with the previous study conducted by Drali et al. [5], reporting a dual infestation with clades A and D among 14 of 37 (37.8%) infested people in DR Congo. It would be interesting to determine whether or not there is evidence of gene exchange and recombination between these different clades or whether lice are living in sympatry. Nevertheless, there was no dual infestation by both clades A and E, nor by clades D and E. Such double infestation may be due to the several infestation events. Multiple infestations may also facilitate the transmission of louse-borne pathogens. The dissemination of lice is also linked to globalization which is led by a significant dynamic of the world’s population [35].

So far, only body lice are considered as vectors of pathogenic bacteria [41, 42]. However, the role of head lice as vectors of infectious diseases is currently more and more discussed. Indeed, studies have reported the presence of DNA of pathogenic bacteria, such as *B. quintana, B. recurrentis*, *Y. pestis* and *C. burnetii* in head lice [5, 6, 12, 17, 20]. Here, we screened 181 head lice collected for several pathogenic bacteria. In our study, only *Acinetobacter* species was found and *A. baumannii* was the most prevalent. This is consistent with previous studies that showed that *A. baumannii* is the most abundant species found in head and body lice [20]. Another study conducted in Congo (Brazzaville) on lice of the pygmy populations found 10.4% of *A. baumannii*, as well as several other *Acinetobacter* species such as *Acinetobacter junii* (18.31%), *Acinetobacter ursingii* (14.35%), *Acinetobacter johnsonii* (9.40%), *Acinetobacter schindleri* (8.41%), *Acinetobacter nosocomialis* (3.18%), *Acinetobacter lwoffii* (4.45%), and *Acinetobacter towneri* (1.98%) [12]. Among *Acinetobacter* species, *A. baumannii* is the most important species, observed worldwide and involved in hospital-acquired infections, including epidemics that are a real challenge for public health. Currently, *A. baumannii* is considered a pathogen responsible for nosocomial infections, but also community acquired infections and infections related to war and natural disasters, such as war wounds among Iraqi and Afghan soldiers [42–45]. Our study is the first to describe *A. soli*, *A. pittii* and *A. guillouiae* in human lice. Unlike *A. guillouiae* which is an environmental species rarely associated with infections, *A. soli* and *A. pitti* have been isolated from clinical samples and are associated with carbapenem resistance [46, 47]. We also detected a potential new species, provisionally referred here as “*Candidatus* Acinetobacter pediculi” in human lice. In the phylogenetic tree (Fig. 3), the sequence of this potential new species forms a separate and well-supported (bootstrap value of 94) branch, that clustered together within the clade that contains *A. guillouiae*. However, the detection of this potential new species has its limitations, as not all previously described species of *Acinetobacter* are already molecularly characterized, so the identification of a new genotype variant may be the re-discovery of an old incompletely characterized species. Further studies are needed to confirm if this new genetic variant represents a new species. Furthermore, despite the fact that several studies have demonstrated widespread infection among lice with several species of *Acinetobacter*, suggesting that lice could be a preferential host for these bacteria, the association between *Acinetobacter* and *Pediculus* lice is still poorly understood [12, 18, 19]. For example, it is still unknown how these lice acquire and transmit *Acinetobacter* infections to their human hosts. Several reports have suggested that the infection could occur after the ingestion of infected blood meal from infected individuals [25, 48, 49]. Indeed, an experimental study demonstrated that the body louse, feeding on bacteremic rabbits, is able to acquire and maintain a persistent life-long infection with *A. baumannii* and *A. lwoffii* and disseminate viable organisms in their feces [49]. The transmission of these infections to humans occurs by contamination of bite sites, microlesions of the skin and mucous membranes with the feces or crushed bodies of infected lice [41]. Further studies are needed to investigate the specificities of the associations between lice and *Acinetobacter* infections.

## Conclusions

In conclusion, we highlighted the presence of clade E head lice in Central Africa. The more prevalent head lice clades in DR Congo were clades A and D. Several *Acinetobacter* species were detected, including one potential new one. More importantly, these ubiquitous opportunistic bacteria reservoirs and their potential involvement in human infections should be put under surveillance.

## Additional file

**Additional file 1.** Geographical occurrences and frequencies of cytb haplotypes of human head and body lice.

## Data Availability

All data generated or analyzed during this study are included in this published article and its additional file. The newly generated sequences were submitted to the GenBank database under the accession numbers MH230910-MH230920 for *Acinetobacter* spp. and MH230921-MH230928 for cytochrome *b* sequences.

## Abbreviations

DR Congo: Democratic Republic of the Congo
Cytb: Cytochrome b
qPCR: quantitative real-time PCR
mtDNA: mitochondrial DNA
